# Excellent PROM results after fast-track hip and knee arthroplasty with no postoperative restrictions: a cohort study validation of fast-track surgery without postoperative restrictions

**DOI:** 10.1186/s12891-022-05276-y

**Published:** 2022-04-05

**Authors:** Aksel Paulsen, Ane Djuv, Jarle Ludvigsen, Ingvild Dalen

**Affiliations:** 1 Department of Orthopaedic Surgery, Stavanger University Hospital, Stavanger, Norway; 2 Department of Public Health, Faculty of Health Sciences, University of Stavanger, Stavanger, Norway; 3 Department of Research, Stavanger University Hospital, Stavanger, Norway

**Keywords:** Arthroplasty, Fast-track, Hip, Knee, No postoperative restrictions, Patient reported outcome measures

## Abstract

**Background:**

Fast-track hip and knee arthroplasty (HA and KA) has been increasingly common over the last decade. In the same time period, there was a strong trend toward less restrictive mobilization. However, few reports have been published on combining these novel programs while measuring the postoperative results by patient-reported outcome measures (PROMs). Descriptions of fast-track surgery programs and their results are warranted.

**Methods:**

The aim of this retrospective cohort study was to examine if it is possible to achieve excellent PROM results for hip and knee arthroplasty patients in a fast-track pathway without postoperative restrictions. During 2014–2017, the stepwise introduction of a PROM program was implemented at Stavanger University Hospital for all scheduled HA and KA patients, with preoperative assessments and postoperative follow-ups at the outpatient clinic. Standardized information with a focus on early mobilization and no postoperative restrictions was also initiated for the same patients. The generic EuroQol questionnaire (EQ-5D) and either the Hip or Knee disability/injury and Osteoarthritis Outcome Score (HOOS or KOOS) were used.

**Results:**

PROM response rates varied from 80 to 99%. The median (interquartile range) change from preoperative to one-year postoperative results were as follows for HA and KA patients, respectively: pain, 55 (43–68) and 47 (31–61); other symptoms, 50 (40–65) and 36 (19–50); function in daily living, 54 (41–65) and 44 (31–55); function in sports and recreation, 56 (38–75) and 40 (15–64); joint-related quality of life, 69 (50–81) and 56 (38–75). The length of stay (LOS) was reduced by 1.9 days (mean), corresponding to a 40% reduction for HA patients and a 37% reduction for KA patients.

**Conclusions:**

We found excellent PROM results after fast-track HA and KA with no postoperative restrictions. We believe that a fast-track program focusing on mobilization without any postoperative restrictions is superior for most patients, but further comparative studies are warranted.

**Supplementary Information:**

The online version contains supplementary material available at 10.1186/s12891-022-05276-y.

## Background

### Fast-track

Fast-track surgery, also called the enhanced recovery surgical program, implies a coordinated perioperative approach aimed at reducing surgical stress and facilitating postoperative recovery [1]. The introduction of fast-track total knee arthroplasty (KA) and total hip arthroplasty (HA) has been a tremendous success [2, 3], and the concepts are well established, including optimized logistics and evidence-based treatment [4, 5], leading to a reduced postoperative length of stay (LOS) [6], lower mortality and morbidity rates [3, 7, 8], and greater patient satisfaction [8]. Fast-track concepts can only be successfully implemented through close interdisciplinary teamwork. Preoperatively, a patient seminar can help to better prepare patients for surgery; while postoperatively, early mobilization and pain treatment play central roles [9]. An effective follow-up regimen with a focus on early mobilization can reduce postoperative pain and can also result in a shorter LOS [10]. Some claim all patients are eligible for fast-track surgery, and the fast-track concept should be standard at all joint replacement facilities [1, 11].

### Postoperative restrictions

Activity restrictions after HA and KA have been common [12], and restrictions following HA for at least 3 months have been widely practiced [13]. There are reports showing that departments with recommendations for activity restrictions have fewer dislocations than departments without restrictions [14], but other studies find that fewer lifestyle restrictions and precautions following HA seem to lead not to worse dislocation rates but to earlier and better resumption of activities and greater patient satisfaction, and these results appear to hold up for various surgical approaches [15–17]. Hip precautions may unnecessarily exacerbate patient anxiety and fear of dislocation following HA [18]. Emerging evidence suggests that these postoperative restrictions are not necessary [15, 19–21].

### Patient-reported outcome measures (PROMs)

Although joint arthroplasty is a successful intervention in terms of prosthesis survival [22], 7–34% of patients have persistent pain or otherwise do not benefit from the operation [23]. Patient-reported outcome measures (PROMs) allow us to examine the patient perspective on joint arthroplasty, have been used increasingly in both orthopedic surgery and health services in general over the last decade [24, 25] and are increasingly used in national arthroplasty registries [26]. PROMs are therefore highly relevant outcome measures after arthroplasty [27].

### Aim

Fast-track joint arthroplasty and minimal postoperative activity restrictions are both increasingly common, but few reports on combining these programs have been published. We aimed to assess the combined programs’ postoperative results from a patient perspective by using PROMs.

## Methods

### Patients

All patients over 18 years of age admitted for elective hip or knee arthroplasty (HA or KA) at the Department of Orthopaedic Surgery at Stavanger University Hospital (SUH) during 2014–2018 who underwent regular pre- and postoperative assessments were included in the fast-track regimen. All patients meeting these inclusion criteria were encouraged to participate in PROM data collection. Patients with cognitive, language, or other impairments preventing PROM completion were excluded from the present study.

### Fast-track regimen

In 2014, we gradually introduced a fast-track regimen consisting of standardized pain medication and standardized information with a focus on mobilization with no postoperative restrictions, postoperative early control at outpatient clinics and PROMs for all scheduled HA and KA patients. Both HA and KA patients were allowed full weight bearing immediately after the operation and had no activity restrictions of any kind. A preoperative PROM program and preoperative assessment at the outpatient clinic were introduced later the same year, and in 2017, we introduced a one-year postoperative PROM program and one-year postoperative control visit at the outpatient clinic for all HA and KA patients. In our fast-track system, the patient was admitted on the day of surgery, and an admission note was made at the outpatient clinic prior to admission. Two weeks before surgery, a full journal recording was made, including blood samples, ECG, X-rays of the joint and information from the operating surgeon. Preoperatively, the patients were assessed, received information and provided PROMs. The standardized premedication consisted of paracetamol 2 g, celecoxib 400 mg and fortecortin 12 mg. If celecoxib or fortecortin was contraindicated, it was replaced by oxycodone 10 to 20 mg. Postoperative mobilization starts as soon as possible, depending on the anesthesia (within 3–6 h). Spinal anesthesia was primarily performed, exceptionally general anesthesia, based on the anesthetists decision. On the first postoperative day, all patients were mobilized guided by a physiotherapist. Our discharge criteria were as follows: ability to get in and out of bed independently, get dressed, go to the toilet, walk with crutches in stairs independently, and no wound leakage. The standardized postoperative pain treatment consisted of paracetamol 1 g × 4, celecoxib 200 mg × 2 and tramadol 50 mg × 3. Six to eight weeks postoperatively, the patients underwent the same assessment as preoperatively, in addition to an X-ray examination. One year postoperatively, the assessment was repeated with the same PROM questionnaires and clinical examination. Only patients requiring additional follow-up at one year (patients with pain from the operated area or subjective instability) were scheduled for a one-year postoperative X-ray examination.

Follow-up within the fast-track regimen consists of the following:

1. Standardized written and oral information with a focus on mobilization and no postoperative restrictions at the early postoperative follow-up visit.
2. Standardized pain medication regimens to minimize opioid use at the early postoperative follow-up visit.
3. Registration of the postoperative course (discharge to their own home, admission to rehabilitation institution, physical therapy, sick leave) at the early postoperative and one-year follow-up visits.
4. An examination of the postoperative status (hip/knee pain, squeaking, leg length discrepancy, knee extension/flexion, knee stability, timed up & go (TUG) test, Trendelenburg sign test, hip abduction test) was performed at the early postoperative and one-year follow-up visits.
5. Registration of postoperative complications (hip dislocation, deep venous thrombosis, nerve damage, superficial infection, deep infection, cerebral insult, pneumonia, urinary tract infection, allergic reaction, other postoperative complications, readmission, death) at the early postoperative and one-year follow-up visits.
6. PROMs: The EQ-5D and Hip or Knee disability/injury and Osteoarthritis Outcome Score (HOOS or KOOS) at the early postoperative and one-year follow-up visits. The preoperative EQ-5D was included late in the introduction of the fast-track regimen.
7. Patient satisfaction (postoperative general health, operative result, change in general health, change in joint symptoms) at the early postoperative and one-year follow-up visits.

### PROMs

All patients provided two different PROMs: one generic, i.e., the EQ-5D, and one condition-targeted, i.e., the HOOS or KOOS.

The EQ-5D is a generic health-related quality of life (QoL) outcome measure that covers five dimensions: mobility, self-care, usual activities, pain/discomfort and anxiety/depression [28]. One of five levels of severity is chosen for each dimension, as this 5L-version is more responsive in our patient group [29]. Patients also rated their current state of health from 0 (‘worst imaginable’) to 100 (‘best imaginable’) on a visual analogue scale (VAS). The EQ-5D generates two values for QoL, one from the patient’s perspective (the EQ-VAS; ‘current state of health’) and the other from a societal perspective, the EQ-5D Index (a health profile that can be made into a global health index with a weighted total value for health-related (QoL)), which represents the patients’ description of their own health and how this health state is perceived by the general population. License was obtained from the EuroQol Group (http://www.euroqol.org/), license agreement number: 152277.

The HOOS is hip-specific and includes 40 items [30]. The KOOS is knee-specific and includes 42 items [31]. Both have five subscales: pain, other symptoms (symptoms), activity/function in daily living (ADL), function in sports and recreation (sports) and joint-related QoL. The sum scores of the subscales range between 0 and 100, with higher scores being better. The HOOS and KOOS do not require any license and can be used free of charge. User guides and scoring manuals are available at http://www.koos.nu/index.html. The HOOS and EQ-5D have been shown to be feasible for use in large-scale studies of HA [32].

### Definition of success –MCII

A statistically significant change in the PROM score does not necessarily represent a clinically important improvement. Since it can be problematic to interpret changes in scores in a clinically meaningful way [33], different cutoff points can be determined based on anchor questions, i.e., items that establish an external patient-reported reference between the PROM change scores and the patients’ health situation. The minimal clinically important improvement (MCII) is a PROM change score value defined as the minimal change representing a clinically important improvement from the patient’s perspective [34]. We compared each patient’s score to published MCII cutoffs [27, 35, 36]. When more than 75% of the patients achieved joint-specific PROM change scores in all of the most relevant domains (pain, symptoms, ADL and QoL) at or above the MCII cutoff, we defined the result as excellent.

#### LOS

We examined LOS in the study period and at the start of the organized efforts to reduce LOS at SUH, as this has been a long-term goal since 2007. Fifty randomly selected patients were included in the retrospective assessment to illustrate the change in LOS from 2007 to 2018.

### Statistics

All data analyses were performed using SPSS (SPSS, Inc., Chicago, IL, USA). Descriptive statistics of categorical variables are presented as counts and percentages. Due to skewed distributions, continuous variables are presented as medians and interquartile ranges (IQRs). Parametric statistics, i.e. means, standard deviations (SD), were performed as supplementary analyses to facilitate comparison to previous published arthroplasty registry data.

### Ethics

The study was submitted for registration to the Regional Committee for Ethics in Medical Research (2018/268) and considered a follow-up quality-control study under the jurisdiction of the local data protection officer. Due to the use of only anonymous data from patient journals, there was no need for informed written consent from patients, and the study was approved by the local data protection officer (journal number 6/2018). The patients will not receive any treatment in addition to our routine medical treatment.

## Results

### Description of the study population

In total, we have PROM data for 1508 surgical procedures in 1393 unique patients; 1284 patients contributed data for one surgical procedure; 103 patients contributed data for two surgical procedures; and six patients contributed data for three surgical procedures. When looking at hips and knees separately, we have data for 917 hip replacements in 876 unique (though they may have also undergone knee replacement) patients and 591 knee replacements in 517 unique patients. Patient characteristics are listed in Table 1.

**Table 1:** Patient characteristics

	Hip arthroplasty (*n* = 917)	Knee arthroplasty (*n* = 591)
Male sex	315 (34%)	249 (42%)
Age at time of surgery	71 (63, 77) [18, 94]	70 (63, 76) [37, 90]
Left side	413 (45%)	294 (50%)
ASA = 0	47 (5%)	17 (3%)
ASA = 1	124 (14%)	63 (11%)
ASA = 2	570 (62%)	383 (65%)
ASA = 3	174 (19%)	128 (22%)
ASA = 4	2 (0.2%)	-

Data are presented as the count (percent) or median (interquartile range; IQR) [full range]

### PROM results

The response rate for the different PROMs varied from 80 to 99%. The preoperative, postoperative and one-year follow-up scores, as well as changes in scores between the different assessment times, are presented for the HA and KA patients in Tables 2 and 3, respectively. The median (IQR) preoperative HOOS symptom score was 35 (25–45) and improved to 90 (80–100) at the one-year follow-up and was the HOOS score with the least improvement; i.e., all other PROMs showed greater absolute improvement for these HA patients. The median (IQR) preoperative HOOS QoL score was 19 (6–31) and improved to 94 (75–100) at the one-year follow-up and was the HOOS score with the highest improvement. Similarly, the KOOS score with the least absolute improvement (KOOS symptoms) improved from 50 (36–61) to 89 (79–96) at the one-year follow-up. The KOOS QoL improved the most, from 19 (11–25) to 75 (56–94) at the one-year follow-up.

**Table 2:** Pre- and postoperative PROM scores and change scores for hip arthroplasty patients

PROM	n	Preop	n	Postop	n	One-year FU	n	Change preop to postop	n	Change preop to one-year FU
HOOS pain	682	35 (25, 45)	678	90 (76, 98)	383	98 (88, 100)	499	51 (38, 63)	284	55 (43, 68)
HOOS symptoms	686	35 (25, 45)	680	85 (75, 95)	385	90 (80, 100)	502	50 (35, 60)	287	50 (40, 65)
HOOS ADL	683	37 (27, 46)	679	88 (77, 96)	385	94 (84, 100)	503	49 (37, 60)	285	54 (41, 65)
HOOS sports	674	19 (6, 31)	662	69 (50, 88)	384	81 (63, 100)	481	50 (31, 69)	284	56 (38, 75)
HOOS QoL	683	19 (6, 31)	680	81 (63, 100)	385	94 (75, 100)	500	56 (44, 75)	287	69 (50, 81)
EQ-5D Index	94	0.59 (0.32, 0.76)	666	0.89 (0.78, 1.00)	380	0.94 (0.83, 1.00)	29	0.29 (0.15, 0.45)	0	-
EQ-VAS	93	60 (50, 73)	668	80 (60, 90)	381	80 (65, 90)	29	20 5, 38)	0	-

The results are presented as the median (interquartile range; IQR). FU: Follow-up. Preop: Preoperative. Postop: Postoperative

**Table 3:** Pre- and postoperative PROM scores and change scores for knee arthroplasty patients

PROM	n	Preop	n	Postop	n	One-year FU	n	Change preop to postop	n	Change preop to one-year FU
KOOS pain	439	39 (25, 47)	428	69 (53, 83)	243	92 (78, 100)	317	28 (14, 44)	182	47 (31, 61)
KOOS symptoms	445	50 (36, 61)	427	75 (61, 82)	243	89 (79, 96)	324	18 (4, 32)	180	36 (19, 50)
KOOS ADL	439	42 (31, 52)	426	75 (59, 89)	241	91 (74, 97)	321	29 (16, 46)	178	44 (31, 55)
KOOS sports	434	10 (0, 20)	397	30 (15, 50)	241	50 (25, 75)	305	20 (5, 35)	180	40 (15, 64)
KOOS QoL	446	19 (11, 25)	427	63 (44, 75)	242	75 (56, 94)	325	38 (19, 57)	181	56 (38, 75)
EQ-5D Index	62	0.64 (0.45, 0.78)	410	0.86 (0.78, 0.94)	239	0.90 (0.78, 0.94)	14	0.13 (0.04, 0.32)	0	-
EQ-VAS	60	60 (46, 70)	411	75 (62, 85)	239	80 (55, 90)	14	23 (0, 40)	0	-

The results are presented as the median (interquartile range; IQR). *FU* Follow-up, *Preop* Preoperative, *Postop* Postoperative

The corresponding results of parametric statistical analysis are given in Supplementary Tables S1 and S2. The percentage of patients achieving the MCII cutoff for the different subscales is given in Table 4. Eighty-seven percent of HA patients and 79% of KA patients achieved joint-specific PROM change scores for all of pain, symptoms, ADLs and QoL at or above the MCII cutoff.

**Table 4:** MCII cutoff and percent of patients achieving cutoff

	MCII cutoff (Reference)	Percent of patients achieving cutoff
HOOS Pain	24 [35]	91%
HOOS Symptoms	20 [35]	93%
HOOS ADL	14 [35]	94%
HOOS QoL	25 [27, 35]	92%
KOOS Pain	18 [36]	87%
KOOS Symptoms	7 [36]	93%
KOOS ADL	16 [36]	89%
KOOS QoL	17 [36]	89%

#### LOS

Data on LOS are available as metadata from our hospital’s journal systems since 2012. Data prior to this are available through manual searching of the journal systems. A retrospective assessment of LOS in 30 consecutive THAs and 20 TKAs from June 2007 and 30 consecutive THAs and 19 TKAs from November 2007 showed that LOS for HA patients was reduced from 17 days (mean)/15 days (median) to 11 days (mean)/10 days (median). The LOS for KA patients in the same period was reduced from 13 days (mean)/13 days (median) to 11 days (mean)/9 days (median). The LOS changed from 4.8 days (mean) for HA patients and 5.1 days (mean) for KA patients in 2013 to 2.9 (mean) for HA patients and 3.2 days (mean) for KA patients in 2018, representing LOS before and after introduction of the fast-track regimen.

## Discussion

We found excellent PROM results after HA and KA in our fast-track program with no postoperative restrictions.

Berg et al. examined 1-year PROM results in HA and KA fast-track programs in a large Swedish observational study and found that fast-track programs seem to be at least as good as conventional care [37]. In their article, there was no information on the use of postoperative restrictions, but based on another study, > 60% of operating centers seemed to have restrictions following hip arthroplasty [38]—the use of postoperative restrictions following knee arthroplasty is uncertain. In our fast-track program without any postoperative restrictions, the KA patients achieved better 1-year results for all KOOS subscores compared to the Swedish cohort. Too few of our patients answered the EQ-5D at 1 year postoperatively to enable a meaningful comparison.

Anchor-based MCII estimates for the patient group at one year were used when available [35]. For HOOS QoL, the 75^th^ percentile approach cutoff (25) was used instead of the mean score approach (17) [35] due to a lower minimal detectable change (21) [27]. When no anchor-based MCII estimation for the PROMs and patient group at one year was found, two-year cutoff estimations were used [36] to ensure no overestimation of patient-reported results.

### Dislocations and reoperations

In 2009, 87% and 95% of American hip and knee surgeons recommended postoperative activity restrictions (avoiding high-impact activities such as running and jogging) [39]. Unrestricted mobilization following KA might be viewed as less controversial than following HA, but in 2018, 44% of members of the American Association of Hip and Knee Surgeons and Canadian Arthroplasty Society universally prescribed precautions [40]. In the UK, hip precautions are routinely provided by the majority of hospitals [41, 42]. The use of postoperative restrictions following primary HA differs between Nordic countries, with between 19% (in Norway) and 50% (in Denmark) of hospitals performing primary HA allowing mobilization without any restrictions [38]. Over the last five years, there has been a strong tendency toward less restrictive mobilization, but still and especially in Norway, few hospitals allow mobilization without restrictions [38]. It has been a fear that fast-track HA and KA with no postoperative restrictions will lead to more hip dislocations and more reoperations. An increase in the rates of readmission and revision after fast-track implementation has been reported [6]. The revision rate after fast-track HA has been reported to be from 1.4 to 2.9% within 90 days and 2.9–5.5% within 1 year [6]. The removal of mobilization restrictions following primary HA performed with a posterolateral approach did not lead to an increased risk of dislocation within 90 days [43]. Van der Weegen et al. [44] also found that patients may be managed safely with minimal restrictions following posterior-approach HA if combined with the frequent use of larger femoral heads. Crompton et al. found no impact on the dislocation rate or PROM scores following HA performed through a posterior approach, regardless of the use of hip precautions [45]. We also did not find indications of an increased revision rate due to the HA/KA management at SUH: the relative risk for revision after HA at SUH from 2007–2018 compared to all other Norwegian hospitals was 0.77 (95% CI: 0.64–0.93), and SUH had a lower percentage of revisions due to hip dislocation than the national mean [46]. The relative risk for revision after KA at SUH from 2008–2018 compared to all other Norwegian hospitals was 0.82 (95% CI: 0.64–1.05) [46]. There is no evident change in revision of hip or knee arthroplasties due to the combined enhanced recovery program (supplementary Table S1, S2). A possible change in HA patients could be attributed to change in surgical approach (in 2013 almost exclusively direct lateral, changed to 20% anterolateral, 25% posterior and 55% direct lateral in 2016, to almost exclusively posterior approach in 2020), change in fixation (in 2013 almost exclusively reverse hybrid, in 2020 20% hybrid, 10% reverse hybrid, < 10% cemented and > 50% uncemented), different components, different head size (in 2013 100% 28 mm, in 2020 > 30% 36 mm and > 60% 32 mm) or different comorbidity level of patients (in 2013 > 10% the American Society of Anesthesiologists physical status classification (ASA) 1, < 70% ASA 2, > 20% ASA3, in 2020 < 5% ASA1, > 50% ASA2, < 40% ASA3, < 5% ASA 4) [47].

### Local data compared to arthroplasty registries

Our HA results are comparable to the results from The Norwegian National Advisory Unit on Arthroplasty and Hip Fractures, the Swedish Hip Arthroplasty Registry and the Danish Hip Arthroplasty Register (Supplementary Table S3). The Norwegian national registry contain only 1 year PROM data from 1001 hip arthroplasty patients (37% response rate), SUH did not contribute to these national data due to technical issues. The Swedish Hip Arthroplasty Registry has a good response rate of 81% preoperatively and 82% postoperatively (supplementary Table S3).

Our KA results are comparable to the Swedish published results. 37% of the Swedish patients undergoing a primary operation contributed PROM data [48], and the response rate has increased to 78% in 2019 (supplementary Table S4).

### Limitations and strengths

Several methodological limitations should be taken into consideration when interpreting the results of the present study. When combining different protocol changes (i.e., fast-track surgery and no postoperative restrictions), the effect of each individual change is difficult to examine. On the other hand, when examining a regimen including different concepts, the data represent the combination of concepts, which is most important in clinical practice. Few patients (< 100 in each group) answered the preoperative EQ-5D questionnaire. The response rate for all PROMs was high (≥ 80%) and much higher than the minimal response rate of PROMs published for HA patients [49]. High response rates ensure generalizability and minimize selection bias. A response rate ≥ 80% is usually considered to be adequate and sufficiently representative of the sample studied [50]. We used well-validated PROMs for the patient group, with validated setting feasibility [32]. Standardized format paper PROMs were used. All returned questionnaire forms were scanned electronically using a validated automated form-processing technique [51]. Multiple factors (surgical approach, change in fixation, different components, different head size and different comorbidity level of patients) changed during the introduction of our fast-track pathway, and our study is not aimed at establishing an etiology between fast-track, postoperative restrictions and outcome, but we have shown that it is possible to achieve excellent PROM results in a real-world combined enhanced recovery surgical program without postoperative restrictions.

### Importance

This study shows that patients can have excellent outcomes after HA and KA with no postoperative restrictions in a fast-track setting. Allowing mobilization without restrictions may further improve fast-track programs by speeding up recovery and shifting focus from movement restriction to optimized mobilization. An effective analgesic regimen pre- and postoperatively is important to achieve the best possible early mobilization. By using PROMs in the fast-track setting, it could be possible to reduce the follow-up of patients treated successfully with joint arthroplasty and target patients at risk.

## Conclusion

We found excellent PROM results after HA and KA without any postoperative restrictions in our fast-track program. We believe that a fast-track program with a focus on mobilization and without postoperative restrictions is superior for most patients. Further comparative studies including PROM endpoints and subgroups from the whole range of arthroplasty patients are warranted to examine the influence of postoperative restrictions on outcome.

## Supplementary Information

**Additional file 1.**

**Additional file 2.**

**Additional file 3:** **Table S1.** Number of hip arthroplasties and revision causes, StavangerUniversity Hospital 2013-2020. **TableS2.** Number of knee arthroplasties and revision causes, Stavanger UniversityHospital 2013-2020. **TableS3.** Comparison Registry data – SUH data: Mean (SD) pre- and postoperative PROM scoresfor hip arthroplasty patients. **TableS4.** Comparison Registry data – SUH data: Mean (SD) pre- and postoperative PROMscores for knee arthroplasty patients Comparison Registry data – SUH data: Mean (SD) pre- and postoperative PROMscores for knee arthroplasty patients. 

## Data Availability

All data generated or analysed during this study are included in this published article [and its supplementary information files].

## Abbreviations

HA: Hip Arthroplasty
KA: Knee Arthroplasty
PROM: Patient-Reported Outcome Measures
EQ-5D: EuroQol questionnaire
HOOS: Hip disability and Osteoarthritis Outcome Score
KOOS: Knee injury and Osteoarthritis Outcome Score
LOS: Length of stay
SUH: Stavanger University Hospital
TUG test: Timed Up & Go test
QoL: Quality of Life
VAS: Visual Analogue Scale
ADL: Activity in Daily Living
MCII: Minimal Clinically Important Improvement
IQR: InterQuartile Ranges
SD: Standard Deviations
CI: Confidence Intervals
