# Supplementary material for: Excellent PROM results after fast-track hip and knee arthroplasty with no postoperative restrictions: a cohort study validation of fast-track surgery without postoperative restrictions
Source: BMC Musculoskelet Disord. 2022 Apr 5;23:324. doi: 10.1186/s12891-022-05276-y (PMC8985261; doi:10.1186/s12891-022-05276-y)
Supplement: Supplementary file 3 — Additional file 3: Table S1. Number of hip arthroplasties and revision causes, StavangerUniversity Hospital 2013-2020. TableS2. Number of knee arthroplasties and revision causes, Stavanger UniversityHospital 2013-2020. TableS3. Comparison Registry data – SUH data: Mean (SD) pre- and postoperative PROM scoresfor hip arthroplasty patients. TableS4. Comparison Registry data – SUH data: Mean (SD) pre- and postoperative PROMscores for knee arthroplasty patients Comparison Registry data – SUH data: Mean (SD) pre- and postoperative PROMscores for knee arthroplasty patients. [file 12891_2022_5276_MOESM3_ESM.docx]

**Supplementary Material**

Table S1. Number of hip arthroplasties and revision causes, Stavanger University Hospital 2013-2020

| **Year** | **Primary operations** | **Dislocation *** | **Loosening of acetabular component *** | **Loosening of femoral component *** | **Deep infection *** | **Peri- prostthetic fracture *** | **Pain *** | **Osteolysis acet, no loosening *** | **Osteolysis femur, no loosening *** | **Polyethylene wear *** | **Previous Girdlestone *** | **Implant fracture *** | **Gluteal failure *** | **Other *** |
| --- | --- | --- | --- | --- | --- | --- | --- | --- | --- | --- | --- | --- | --- | --- |
|  | n | n | n | n | n | n | n | n | n | n | n | n | n | n |
| 2020 | 346 | 2 | 1 | 0 | 3 | 1 | 0 | 0 | 0 | 0 | 1 | 0 | 0 | 2 |
| 2019 | 396 | 1 | 1 | 2 | 1 | 1 | 0 | 0 | 0 | 0 | 0 | 0 | 0 | 6 |
| 2018 | 414 | 5 | 2 | 1 | 0 | 4 | 0 | 0 | 0 | 0 | 0 | 0 | 1 | 3 |
| 2017 | 390 | 5 | 0 | 1 | 1 | 1 | 1 | 0 | 0 | 0 | 0 | 0 | 0 | 0 |
| 2016 | 345 | 4 | 0 | 2 | 3 | 4 | 1 | 1 | 0 | 0 | 0 | 0 | 0 | 4 |
| 2015 | 354 | 2 | 0 | 1 | 4 | 4 | 0 | 0 | 0 | 0 | 0 | 0 | 1 | 7 |
| 2014 | 337 | 2 | 0 | 2 | 3 | 5 | 0 | 0 | 0 | 0 | 0 | 0 | 0 | 2 |
| 2013 | 387 | 2 | 0 | 3 | 7 | 6 | 0 | 0 | 0 | 0 | 0 | 2 | 0 | 1 |

* Revision causes are not mutually exclusive. More than one reason of revision is possible

2014-2017: stepwise introduction of a fast-track PROM programTable S2. Number of knee arthroplasties and revision causes, Stavanger University Hospital 2013-2020

| **Year** | **Primary operations** | **Dislocation of patella *** | **Dislocation (not patella) *** | **Loose proximal component *** | **Loose distal component *** | **Fracture (near implant) *** | **Pain *** | **Instability *** | **Deep infection *** | **Defect polyethylene *** | **Malalignment *** | **Other *** |
| --- | --- | --- | --- | --- | --- | --- | --- | --- | --- | --- | --- | --- |
|  | n | n | n | n | n | n | n | n | n | n | n | n |
| 2020 | 187 | 0 | 0 | 0 | 0 | 0 | 0 | 0 | 1 | 0 | 0 | 3 |
| 2019 | 221 | 0 | 0 | 0 | 3 | 1 | 0 | 1 | 1 | 0 | 1 | 2 |
| 2018 | 197 | 0 | 0 | 0 | 1 | 1 | 0 | 2 | 0 | 0 | 1 | 1 |
| 2017 | 208 | 0 | 0 | 1 | 0 | 0 | 0 | 1 | 0 | 0 | 1 | 2 |
| 2016 | 231 | 0 | 0 | 0 | 1 | 1 | 2 | 5 | 1 | 0 | 1 | 1 |
| 2015 | 175 | 0 | 0 | 1 | 1 | 0 | 0 | 3 | 4 | 0 | 0 | 3 |
| 2014 | 174 | 1 | 0 | 1 | 2 | 0 | 1 | 0 | 3 | 0 | 1 | 3 |
| 2013 | 158 | 0 | 0 | 1 | 0 | 1 | 3 | 0 | 2 | 0 | 3 | 0 |

* Revision causes are not mutually exclusive. More than one reason of revision is possible

2014-2017: stepwise introduction of a fast-track PROM program

Table S3. Comparison Registry data – SUH data: Mean (SD) pre- and postoperative PROM scores for hip arthroplasty patients

|  | **SUH** | | | | **The Norwegian Arthroplasty Register [1, 2]** | | | | **The Swedish Hip Arthroplasty Registry [3]** | | | | **The Danish Hip Arthroplasty Register [4]** | | | |
| --- | --- | --- | --- | --- | --- | --- | --- | --- | --- | --- | --- | --- | --- | --- | --- | --- |
| **PROM** |  | **Preop** |  | **One-year Postop** |  | **Preop** |  | **One-year Postop** |  | **Preop** |  | **One-year Postop** |  | **Preop** |  | **One-year Postop** |
|  | **n** | **mean**  **(SD)** | **n** | **mean (SD)** | **n** | **mean (SD)** | **n** | **mean (SD)** | **n** | **mean (SD)** | **n** | **mean (SD)** | **n** | **mean (SD)** | **n** | **mean (SD)** |
| HOOS pain | 682 | 35  (15) | 383 | 90  (15) | 2489 | 40 | 151 | 80 |  |  |  |  | 1335 | 44 | 1288 | 89  (16) |
| HOOS symptoms | 686 | 36  (17) | 385 | 87  (15) | 2489 | 39 | 151 | 76 |  |  |  |  |  |  |  |  |
| HOOS ADL | 683 | 36  (16) | 385 | 88  (15) | 2489 | 45 | 151 | 78 |  |  |  |  |  |  |  |  |
| HOOS sports | 674 | 21  (18) | 384 | 76  (24) | 2489 | 28 | 151 | 61 |  |  |  |  |  |  |  |  |
| HOOS QoL | 683 | 21  (15) | 385 | 85  (21) | 2489 | 28 | 151 | 72 |  |  |  |  | 1335 | 31 | 1288 | 80  (22) |
| EQ-5D Index | 94 | 0.53  (0.26) | 380 | 0.87  (0.18) |  |  |  |  |  |  |  |  | 1335 | 0.60 | 1288 | 0.88  (0.16) |
| EQ-5D VAS | 93 | 59  (19) | 381 | 75  (21) | 327 | 54  (21) | 66 | 68  (20) | 24572 | 56  (22) | 28031 | 76  (19) | 1335 | 62 | 1288 | 80  (18) |

Table S4. Comparison Registry data – SUH data: Mean (SD) pre- and postoperative PROM scores for knee arthroplasty patients

|  | **SUH** | | | | **The Norwegian Arthroplasty Register [2]** | | | | **The Swedish Knee Arthroplasty Registry [5]** | | | |
| --- | --- | --- | --- | --- | --- | --- | --- | --- | --- | --- | --- | --- |
| **PROM** |  | **Preop** |  | **One-year Postop** |  | **Preop** |  | **One-year Postop** |  | **Preop** |  | **One-year Postop** |
|  | **n** | **mean**  **(SD)** | **n** | **mean (SD)** | **n** | **mean (SD)** | **n** | **mean (SD)** | **n** | **mean (SD)** | **n** | **mean (SD)** |
| KOOS pain | 439 | 38  (16) | 243 | 84  (20) | 1596 | 44 |  |  | 5150 | 41  (15) | 5150 | 81  (19) |
| KOOS symptoms | 445 | 50  (18) | 243 | 84  (16) | 1596 | 54 |  |  | 5150 | 45  (18) | 5150 | 77  (17) |
| KOOS ADL | 439 | 42  (17) | 241 | 83  (19) | 1596 | 50 |  |  | 5150 | 47  (16) | 5150 | 80  (19) |
| KOOS sports | 434 | 13  (17) | 241 | 51  (28) | 1596 | 17 |  |  | 5150 | 12  (15) | 5150 | 38  (28) |
| KOOS QoL | 446 | 20  (13) | 242 | 73  (24) | 1596 | 25 |  |  | 5150 | 22  (14) | 5150 | 65  (24) |
| EQ-5D Index | 62 | 0.59  (0.22) | 239 | 0.84  (0.17) |  |  |  |  |  |  |  |  |
| EQ-5D VAS | 60 | 57  (19) | 239 | 72    (23) | 1706 | 62  (18) | 491 | 72  (18) | 6279 | 64  (22) |  | 78  (19) |

**References**

1. Norwegian National Advisory Unit on Arthroplasty and Hip Fractures, *Report June 2019*. 2019, Nasjonalt Register for Leddproteser.

2. Norwegian National Advisory Unit on Arthroplasty and Hip Fractures, *Report 2021*. 2021, Nasjonalt Register for Leddproteser.

3. The Swedish Hip Arthroplasty Register, *Annual Report 2018*. 2018.

4. Paulsen, A., *Patient reported outcomes in hip arthroplasty registries.* Dan Med J, 2014. **61**(5): p. B4845.

5. The Swedish Knee Arthroplasty Register, *Annual Report 2020*. 2020.
